# Bone marrow cytology as an adjunct tool for post-mortem interval estimation

**DOI:** 10.3389/fmed.2026.1904465

**Published:** 2026-09-30

**Authors:** Treasa James, Akhilesh Pathak, Ajay Kumar, Manjit Kaur Rana, Priyanka Sharma, Rahul Kaushik, Soumya Swaroop Sahoo

**Affiliations:** 1 Department of Forensic Medicine and Toxicology, All India Institute of Medical Sciences, Bathinda, India; 2 Department of Pathology, AIIMS, Bathinda, India; 3 Department of Community and Family Medicine, AIIMS, Bathinda, India

**Keywords:** autolysis, bone marrow, cytology, forensic pathology, histomorphology, PMI, post-mortem interval, sternal aspiration

## Abstract

**Introduction:**

Estimating the post-mortem interval (PMI) remains challenging, particularly when external indicators are unreliable. Sternal bone marrow may provide a relatively stable substrate for PMI assessment.

**Methods:**

This observational autopsy-based study evaluated sternal bone marrow from 105 medico-legal autopsy cases with known PMI. Giemsa-stained aspirates were graded (1-5) for nuclear and cytoplasmic autolytic changes and grouped into five PMI intervals (<9 h to >27 h).

**Results:**

A highly significant association was observed between PMI and cellular degradation (*p* < 0.001). Early intervals showed preserved morphology, intermediate intervals showed nuclear budding and vacuolation, and advanced intervals demonstrated nuclear fragmentation, debris, and structural loss.

**Discussion:**

Sternal bone marrow cytology demonstrates a significant time-dependent association with PMI and may serve as a low-cost complementary tool during the early to intermediate post-mortem period. Further studies are required to establish its predictive accuracy and reproducibility.

## Introduction

Estimating the postmortem interval (PMI) is one of the most complex and essential tasks in forensic pathology, playing a critical role in reconstructing the chronology of death and aiding medico-legal investigations. It assists in verifying witness statements, narrowing suspect pools, and evaluating alibis within the framework of criminal law (1, 2). Accurate PMI estimation requires understanding the biological changes that occur after death and integrating these findings with investigative data (3, 4, 16).

Various methods have been developed to estimate PMI, including physical, biochemical, autolytic, and entomological approaches (9, 13, 21, 25, 31, 32, 34–39). Physical indicators such as algor mortis, rigor mortis, and livor mortis remain widely used but are influenced by environmental and individual factors, limiting their accuracy (5–8, 28, 42–44).

Biochemical methods, such as vitreous potassium estimation and molecular techniques like RNA degradation analysis, offer improved precision but are constrained by decomposition, contamination, and variability in later intervals (9, 11–14, 17, 31, 38).

Due to these limitations, there is a need for more stable biological substrates. Bone marrow, protected within bone and relatively resistant to autolysis, has emerged as a promising alternative (45–58). Its anatomical insulation reduces the influence of environmental factors, preserving cellular morphology even when other tissues are degraded (12–14, 18, 19, 22–24, 26–30, 33, 40, 41, 67, 68). Histologically, bone marrow exhibits a predictable sequence of postmortem changes, including nuclear condensation, fragmentation, and cytoplasmic vacuolation, which correlate with PMI (19, 22, 27, 30, 33, 40, 41, 59–62, 71–73).

Sternal bone marrow is particularly suitable due to its accessibility, minimal contamination risk, and consistent preservation, even in advanced decomposition or conditions such as drowning (10, 12–14, 64, 65, 75). Its evaluation is technically simple, cost-effective, and feasible in routine forensic practice (10, 14, 15, 64, 65, 74, 75).

Therefore, bone marrow histomorphology represents a reliable adjunct to conventional PMI estimation methods, offering a reproducible and objective approach, particularly in the early to intermediate postmortem period.

## Materials and methods

### Study design and setting

This observational autopsy-based study was conducted from February 2024 to October 2025 after obtaining approval from the Institutional Ethics Committee. Written informed consent was obtained from the legal next of kin prior to inclusion.

### Study population

The study included all medico-legal autopsy cases with a known post-mortem interval (PMI) during the study period.

### Inclusion and exclusion criteria

The study included medico-legal autopsy cases examined during the study period in which the post-mortem interval was known and consent for bone-marrow sampling had been obtained from the legal next of kin. Cases were excluded when neoplastic or other pathological conditions affecting bone or bone marrow were present, as these could independently alter marrow morphology. Cases with sternal trauma were excluded because local injury could interfere with sampling and cytological interpretation. Cases with an unknown PMI were excluded because they could not be allocated to the predefined PMI categories. Cases without consent and cases showing advanced decomposition that precluded adequate morphological assessment were also excluded.

### Sample size

The minimum sample size was calculated using a standard deviation of 14.41 from a previous study by Satish Babu et al. (15), yielding 34 cases. However, to improve validity, all eligible cases were included, resulting in a total sample of 105 cases.

### Bone-marrow sampling and processing

Autopsies were performed using Letulle's technique. Bone marrow was aspirated from the sternum using a 16-gauge needle and 10 mL syringe under aseptic precautions. Smears were prepared on clean slides, air-dried, fixed in methanol for 10 min, and stained with Giemsa. Slides were then washed, dried, and mounted using DPX (Dibutylphthalate plasticizer xylene) mounting medium.

### Microscopic examination

Slides were examined under 10×, 40×, and 100× magnifications. Erythroid, myeloid, and megakaryocytic series were evaluated for nuclear and cytoplasmic morphology, autolysis, and cellular depletion.

### Grading of autolytic changes

Cellular changes were graded using a modified five-grade system adapted from Biradar et al. (3). Nuclear changes were classified as Grade 1, intact morphology; Grade 2, nuclear budding; Grade 3, syncytial nuclear changes; Grade 4, nuclear fragmentation; and Grade 5, complete loss of identifiable nuclear morphology. Cytoplasmic changes were classified as Grade 1, preserved cytoplasm; Grade 2, small cytoplasmic vacuoles; Grade 3, prominent/large cytoplasmic vacuoles; Grade 4, marked degeneration with near-complete cytoplasmic loss; and Grade 5, complete cytoplasmic dissolution. The predominant grade was recorded after repeated evaluation and supervisor verification.

### Data collection

Data were collected using a structured proforma including demographic details, cause of death, and bone-marrow findings. All cases were anonymised and coded before analysis.

### Statistical analysis

Statistical analysis was performed using SPSS version 29.0. Continuous variables were expressed as mean ± standard deviation, and categorical variables as frequency and percentage. Associations were analysed using Chi-square and independent t-test, with *p* < 0.05 considered significant and *p* < 0.001 highly significant.

### Ethical considerations and conflict of interest

The study adhered to ethical guidelines for biomedical research involving human tissue, with confidentiality maintained throughout. The authors declare no conflict of interest.

## Results

The present study included 105 medico-legal autopsy cases in which sternal bone marrow samples were analysed, and cellular changes were graded using the modified five-grade system adapted from Biradar et al. (3). Cases were categorised into five post-mortem interval (PMI) groups: <9 h, 9–15 h, 15–21 h, 21–27 h, and >27 h. Of the 105 cases, 2 (1.9%) were in the <9-hour group, 10 (9.5%) in the 9–15-hour group, 19 (18.1%) in the 15–21-hour group, 26 (24.8%) in the 21–27-hour group, and 48 (45.7%) in the >27-hour group. The >27-hour group included cases with PMI extending up to 3 weeks. Advanced autolytic changes predominated in this group, limiting further morphological differentiation. The study population comprised 72 males (68.6%) and 33 females (31.4%). The mean age of males (43.78 ± 16.01 years) and females (41.10 ± 15.42 years) showed no significant difference (*p* = 0.516). Mean height was significantly higher in males than females (*p* = 0.008), whereas no statistically significant sex-based differences were observed for age (*p* = 0.516) or body weight (*p* = 0.072) (Table 1). These analyses were descriptive and were not designed to assess the independent association of demographic variables with marrow preservation grades. A highly significant association (*p* < 0.001) was observed between PMI and cellular changes across erythroid, myeloid, and megakaryocytic lineages (Table 2). In early post-mortem intervals, all cases nuclear and cytoplasmic morphology was preserved in all cases whereas with increasing PMI there was a progressive decline in cellular integrity, and beyond 27 h only 12.5% of cases retained identifiable structures while 87.5% showed complete loss. Erythroid cells showed early nuclear preservation followed by rapid karyolysis, while cytoplasmic remnants persisted longer as eosinophilic debris. Myeloid cells exhibited simultaneous nuclear and cytoplasmic degradation, indicating uniform autolysis, whereas megakaryocytes demonstrated relatively prolonged structural preservation but showed marked loss after 27 h. Grading analysis revealed a clear chronological sequence of autolytic changes (*p* < 0.001) (Tables 3, 4), with Grade 1 changes in <9 h showing intact nuclei and cytoplasm, Grade 2 changes in 9–15 h characterised by nuclear budding and small vacuoles, Grade 3 changes in 15–21 h showing syncytial nuclear transformation and large vacuoles, Grade 4 changes in 21–27 h with nuclear fragmentation and near-complete cytoplasmic loss, and Grade 5 changes beyond 27 h indicating complete structural disintegration. Overall, cellular preservation was good up to 21 h, followed by a transitional phase between 21 and 27 h, and complete degradation beyond 27 h, establishing a consistent time-dependent autolytic pattern. These findings demonstrate a statistically significant association between increasing PMI categories and progressive bone marrow autolytic changes, particularly within the first approximately 27–30 h. Representative cytological changes are shown in Figures 1–5.

**Table 1:** Comparison of demographic variables (age, height, and weight) between male and female post-mortem cases.

Variables	Male (mean ± SD)	Female (mean ± SD)	*p*-value
Age	43.78 ± 16.01	41.10 ± 15.42	0.516
Height	171.42 ± 10.18	165.80 ± 13.57	0.008
Weight	68.78 ± 11.77	66.45 ± 15.45	0.072

**Table 2:** Association of erythroid, myeloid, and megakaryocytic nuclear and cytoplasmic changes with different post-mortem interval groups (*p* < 0.001).

		Post-Mortem Interval					TOTAL	*p*-value
Cellular component	Status	<9 h *n* (%)	9–15 h *n* (%)	15–21 h *n* (%)	21–27 h *n* (%)	>27 h *n* (%)	*n* (%)	
Erythroid (NUCLEUS)	PRESENT	2 (100)	10 (100)	17 (89.5)	26 (100)	6 (12.5)	61 (58.1)	<0.001
	ABSENT	0 (0)	0 (0)	2 (10.5)	0 (0)	42 (87.5)	44 (41.9)	
Erythroid (Cytoplasm)	PRESENT	2 (100)	10 (100)	17 (89.5)	26 (100)	6 (12.5)	61 (58.1)	<0.001
	ABSENT	0 (0)	0 (0)	2 (10.5)	0 (0)	42 (87.5)	44 (41.9)	
Myeloid (Nucleus)	PRESENT	2 (100)	10 (100)	17 (89.5)	26 (100)	6 (12.5)	61 (58.1)	<0.001
	ABSENT	0 (0)	0 (0)	2 (10.5)	0 (0)	42 (87.5)	44 (41.9)	
Myeloid (Cytoplasm)	PRESENT	2 (100)	10 (100)	17 (89.5)	26 (100)	6 (12.5)	61 (58.1)	<0.001
	ABSENT	0 (0)	0 (0)	2 (10.5)	0 (0)	42 (87.5)	44 (41.9)	
Megakaryocyte (Nucleus)	PRESENT	2 (100)	10 (100)	17 (89.5)	26 (100)	6 (12.5)	61 (58.1)	<0.001
	ABSENT	0 (0)	0 (0)	2 (10.5)	0 (0)	42 (87.5)	44 (41.9)	
Megakaryocyte (Cytoplasm)	PRESENT	2 (100)	10 (100)	17 (89.5)	26 (100)	6 (12.5)	61 (58.1)	<0.001
	ABSENT	0(0)	0(0)	2(10.5)	0(0)	42(87.5)	44(41.9)	

**Table 3:** Distribution of nuclear grading (grades 1–5) of bone-marrow cells with respect to post-mortem interval.

Grading Nuclear	Post-Mortem Interval					TOTAL	*p*-value
	<9 h *n* (%)	9–15 h *n* (%)	15–21 h *n* (%)	21–27 h *n* (%)	>27 h *n* (%)		
1	2 (100)	0 (0)	0 (0)	0 (0)	0 (0)	2 (1.9)	<0.001
2	0 (0)	1 (10)	0 (0)	0 (0)	0 (0)	1 (1)	<0.001
3	0 (0)	8 (80)	15 (78.9)	2 (7.7)	0 (0)	25 (23.8)	<0.001
4	0 (0)	1 (10)	4 (21.1)	23 (88.5)	0 (0)	28 (26.7)	<0.001
5	0 (0)	0 (0)	0 (0)	1 (3.8)	48 (100)	49 (46.7)	<0.001
TOTAL	2 (100)	10 (100)	19 (100)	26(100)	48(100)	105(100)	

**Table 4:** Distribution of cytoplasmic grading (grades 1–5) of bone-marrow cells across different post-mortem interval groups.

Grading Cytoplasm	Post-Mortem Interval					TOTAL	*p*-value
	<9 h *n* (%)	9–15 h *n* (%)	15–21 h *n* (%)	21–27 h *n* (%)	>27 h *n* (%)		
1	2 (100)	0 (0)	0 (0)	0 (0)	0 (0)	2 (1.9)	<0.001
2	0 (0)	1 (10)	0 (0)	0 (0)	0 (0)	1 (1)	<0.001
3	0 (0)	8 (80)	15 (78.9)	2 (7.7)	0 (0)	25 (23.8)	<0.001
4	0 (0)	1 (10)	4 (21.1)	23 (88.5)	0 (0)	28 (26.7)	<0.001
5	0 (0)	0 (0)	0 (0)	1 (3.8)	48 (100)	49 (46.7)	<0.001
TOTAL	2 (100)	10 (100)	19 (100)	26(100)	48(100)	105(100)	

**Figure 1:**
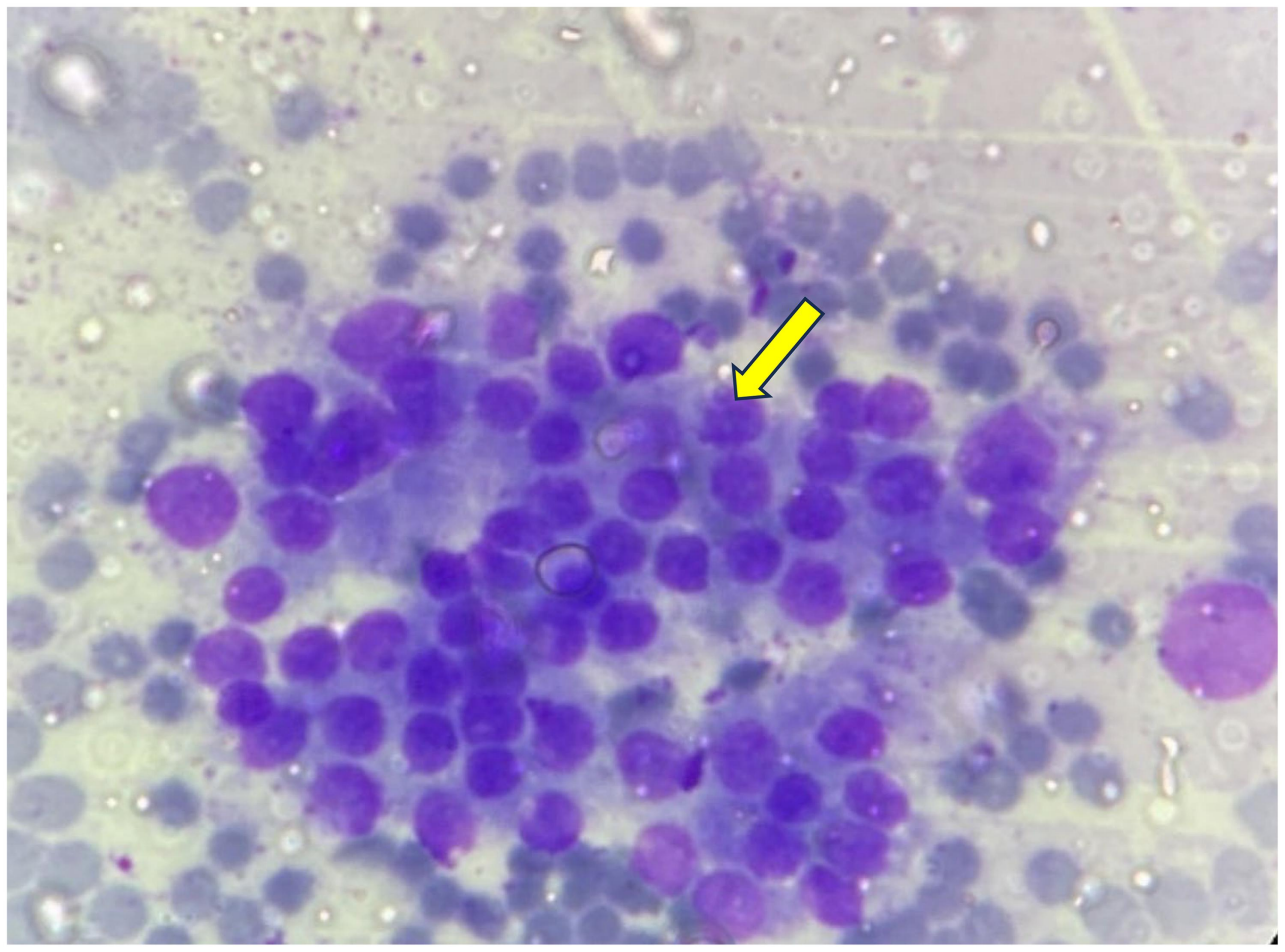
Low-power(10×) figure of Giemsa-stained sternal bone marrow aspirate, showing nuclear changes in the form of syncytium and fragmentation (arrow) of nucleus, along with areas of amorphous debris suggestive of progressing autolysis during the mid to late post-mortem interval. (15−21 h, grade 3).

**Figure 2:**
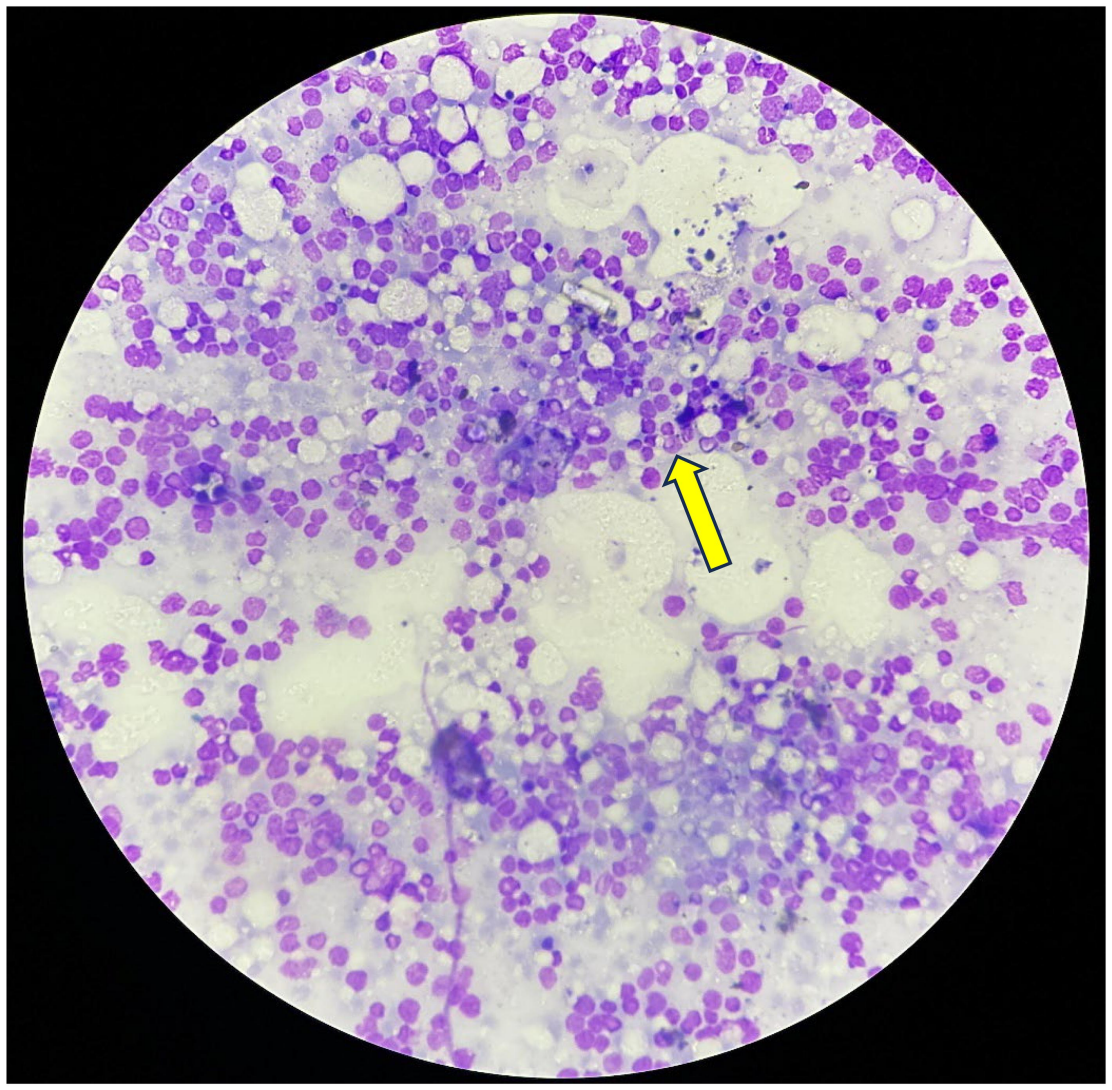
High power (40×) illustration of Giemsa-stained sternal bone marrow aspirate showing moderate cellularity with myeloid and erythroid precursors displaying distinct nuclear morphology. Predominantly minor and a few large cytoplasmic vacuoles (arrow), cytoplasmic irregularities are evident, indicating early autolytic changes, while overall marrow structure remains preserved. (9−15 h, Grade 2).

**Figure 3:**
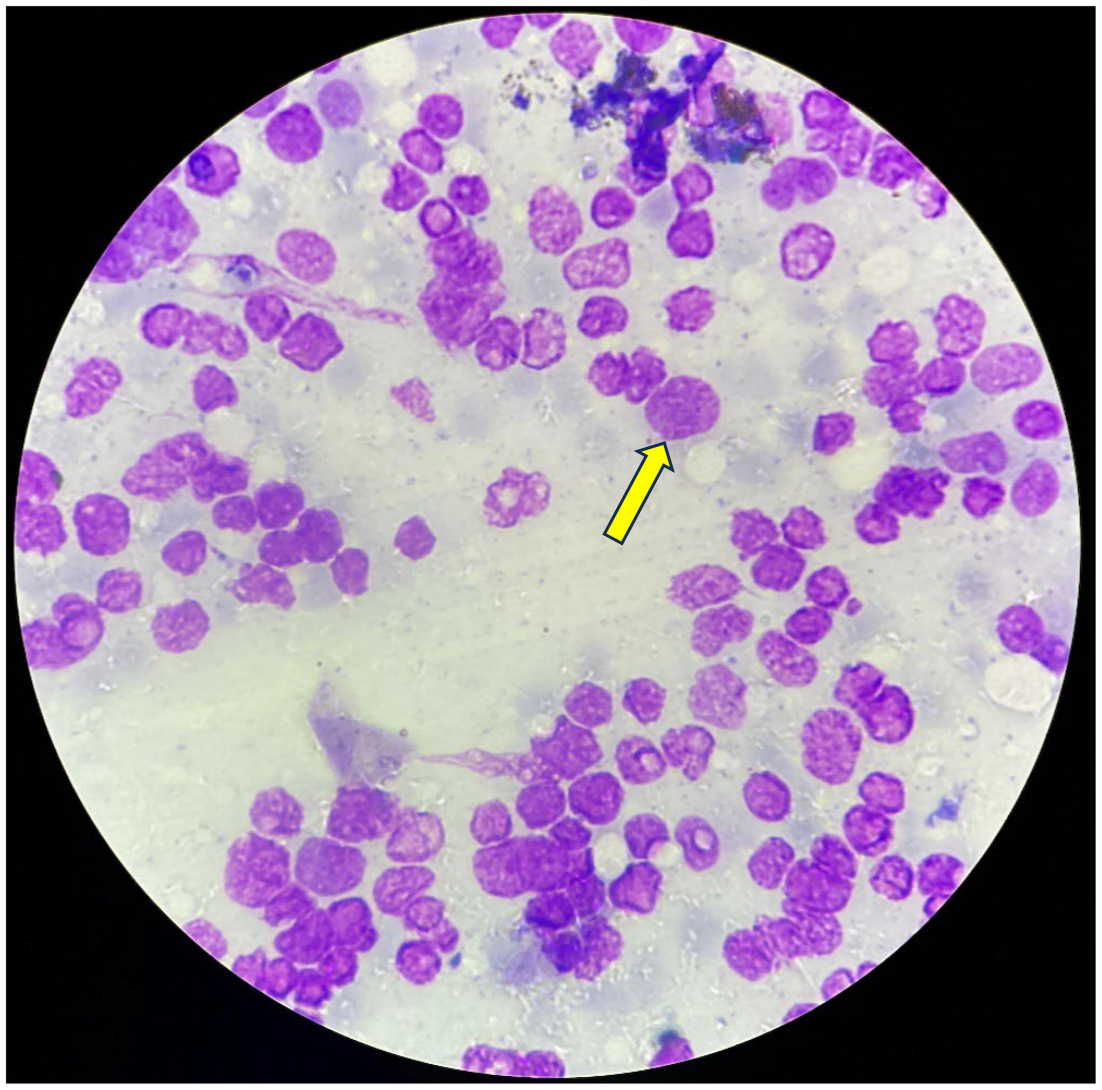
Low-power(10×) image of Giemsa-stained bone marrow aspirate showing markedly reduced cellularity with widespread cytoplasmic degeneration and nuclear loss (arrow). The smear demonstrates scattered cellular debris and pale eosinophilic bac kground material, consistent with advanced autolysis at a late post-mortem interval. (>27 h, Grade 5).

**Figure 4:**
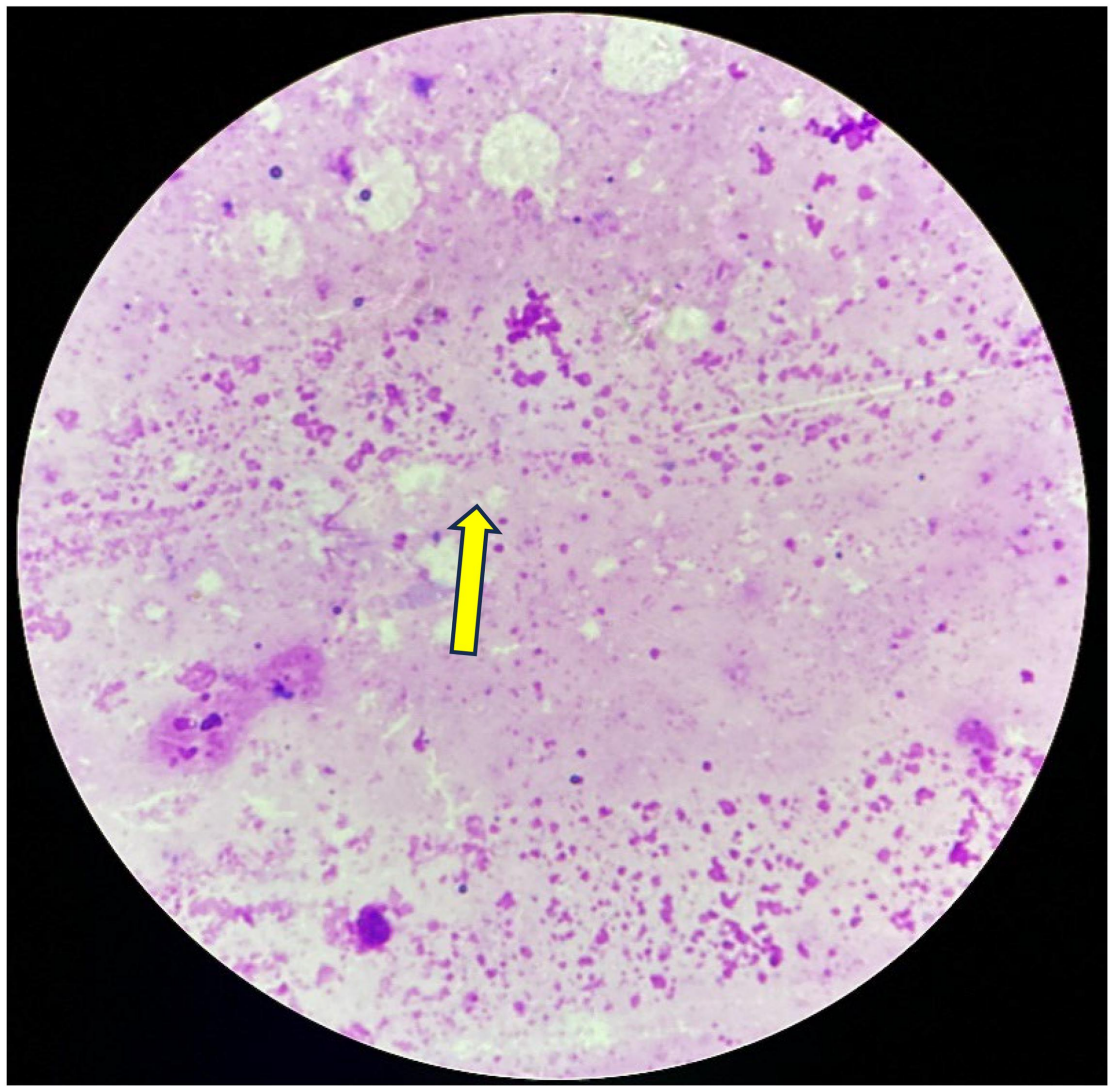
Low-power(10 ×) image of Giemsa stained bone marrow aspirate showing markedly reduced hematopoietic cellularity with extensive cytoplasmic breakdown and nuclear dissolution(arrow). The smear demonstrates clusters of amorphous debris and pale eosinophilic background material, consistent with late post-mortem autolysis and advanced degenerative changes. (>27 h, Grade 5).

**Figure 5:**
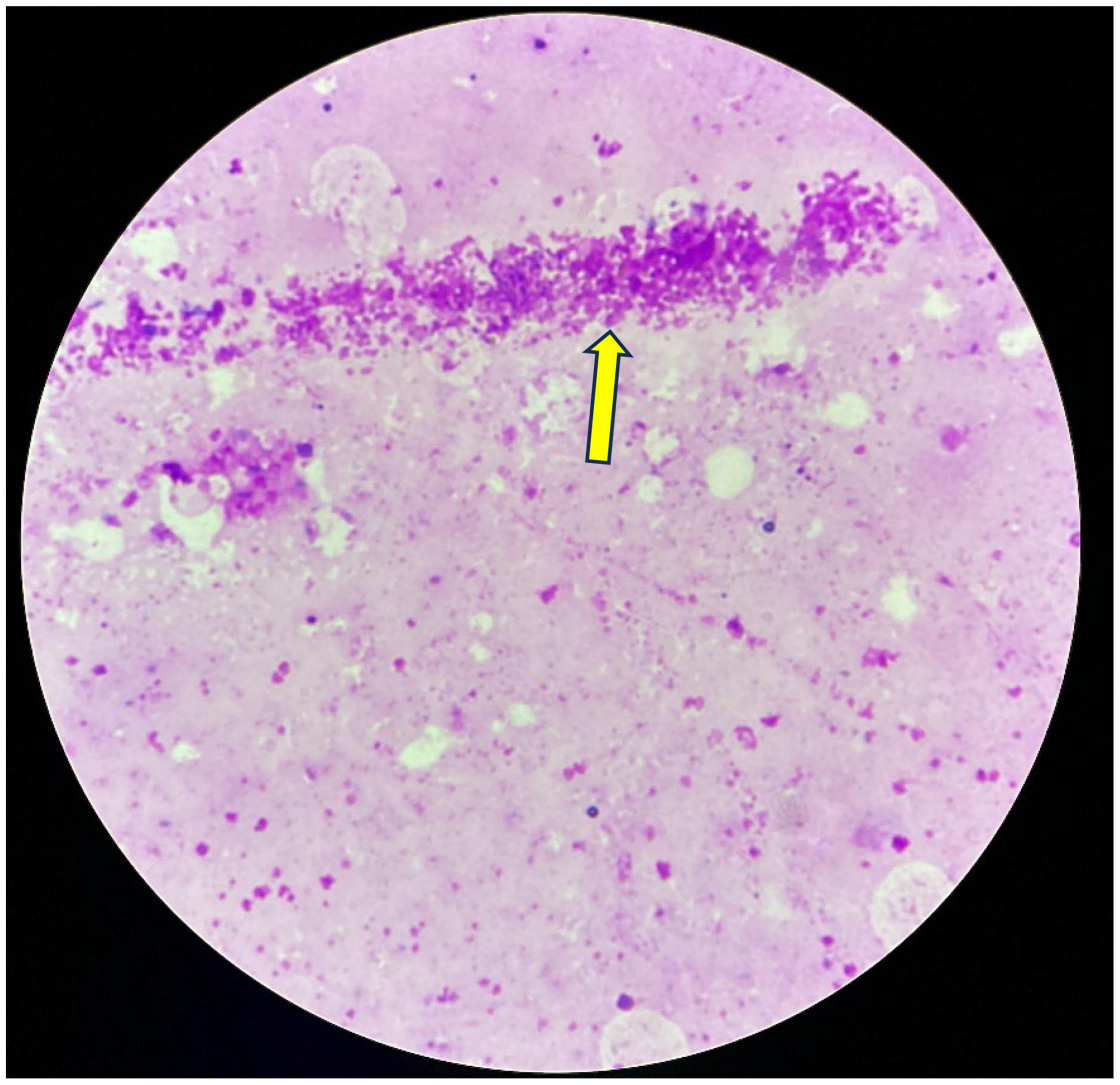
Low-power (10×)image of Giemsa stained bone marrow aspirate showing almost complete disintegration of hematopoietic architecture with prominent clusters of coalescing fat lobules(arrow) and amorphous eosinophilic debris with scattered erythroid cells (>27 h, grade 5).

## Discussion

The estimation of post-mortem interval (PMI) remains one of the most challenging aspects of forensic practice (16, 21, 25, 32, 39). The present study evaluated bone marrow histomorphology as a tool for PMI estimation by analysing 105 medico-legal autopsy cases and correlating nuclear and cytoplasmic changes with defined PMI intervals. The findings demonstrate that bone marrow undergoes a predictable and statistically significant sequence of autolytic changes, confirming its utility as a reliable substrate for PMI estimation, particularly in the early to intermediate post-mortem period (up to approximately 27 h).

Nuclear changes progressed from intact morphology (Grade 1) to budding (Grade 2), syncytial changes (Grade 3), fragmentation (Grade 4), and complete loss (Grade 5). Cytoplasmic changes progressed from preserved morphology (Grade 1) to small vacuoles (Grade 2), prominent or large vacuoles (Grade 3), near-complete cytoplasmic loss (Grade 4), and complete dissolution (Grade 5). These morphologically demonstrable stages enabled objective categorisation of autolysis and facilitated statistical correlation with PMI.

In the earliest interval (<9 h), marrow cells across erythroid, myeloid, and megakaryocytic series showed preserved nuclear and cytoplasmic morphology, consistent with observations by Satish Babu et al. (15) and Biradar et al. (3), who reported minimal autolytic change in early post-mortem periods. As PMI progressed to 9–15 h, early autolytic features such as nuclear budding and cytoplasmic vacuolation became evident, reflecting the onset of intracellular enzymatic degradation. These findings support previous reports that cytoplasmic changes may precede advanced nuclear disintegration (3).

The 15–21 h interval represented a critical transitional phase characterised by syncytial nuclear changes and prominent cytoplasmic vacuolation. This stage corresponds to active autolysis and aligns with findings of Satish Babu et al. (15) and Biradar et al. (3), as well as biochemical observations by Sakr et al. (4), where oxidative stress markers decline significantly. The concurrence of histological and biochemical changes reinforces the biological plausibility of marrow-based PMI estimation.

In the 21–27 h interval, advanced autolytic changes predominated, with nuclear fragmentation and near-complete cytoplasmic loss, indicating extensive cellular breakdown. Beyond 27 h, cellular architecture was largely lost (Grade 5), and although cases in this group extended up to 3 weeks, the advanced degree of autolysis limited further temporal differentiation based on marrow morphology. Similar observations have been reported by Satish Babu et al. (15) and Biradar et al. (3), who noted that nuclear and cytoplasmic details become unrecognisable beyond a certain PMI. This defines a practical upper limit for marrow-based PMI estimation.

Compared to conventional methods such as algor mortis, rigor mortis, and livor mortis, which are influenced by environmental and individual factors, bone marrow offers a relatively protected and stable internal substrate. Studies by Yadav et al. (66) and Giles et al. (69) have demonstrated variability in external decomposition even under controlled conditions, whereas marrow degradation follows a more consistent internal enzymatic pattern. This insulation enhances its reliability, particularly when external indicators are unreliable.

From a practical standpoint, bone marrow aspiration is simple, minimally invasive, and cost-effective, requiring only basic laboratory infrastructure. Unlike entomological or biochemical methods, which require specialised expertise or equipment (4, 70), marrow cytology can be readily incorporated into routine autopsy practice, especially in resource-limited settings.

Previous studies by Tattoli et al. (14) and Roll et al. (11) have highlighted the diagnostic value of bone marrow in post-mortem analysis, while Takahashi et al. (63) demonstrated its role in identifying agonal and pathological processes. The present study extends these findings by establishing a temporal correlation between marrow morphology and PMI, suggesting its dual role in both time-since-death estimation and pathological assessment.

However, certain limitations must be considered. Several factors besides PMI may affect postmortem bone marrow autolysis, including temperature, refrigeration or freezing, cause of death, body habitus, age, and underlying disease (20). These variables were not systematically controlled in the present study and may have contributed to differences in marrow preservation. The potential influence of different causes and mechanisms of death on marrow autolysis was not specifically evaluated in the present study. Future studies should evaluate their independent effects on marrow autolysis.

Overall, the findings support a multimodal approach to PMI estimation. Bone marrow histomorphology demonstrated a statistically significant association with grouped PMI and may provide useful complementary information alongside conventional, biochemical, and entomological methods. However, the present study did not evaluate predictive accuracy, interobserver reproducibility, or external validity. Therefore, marrow morphology should not be interpreted as a stand-alone method for determining PMI. Larger prospective studies incorporating relevant environmental and individual covariates and formal predictive validation are required before specific marrow grades can be used to estimate PMI with defined accuracy.

## Conclusion

This study demonstrates a significant time-dependent association between post-mortem bone-marrow morphology and increasing PMI. Nuclear and cytoplasmic structures showed progressive changes from relatively preserved morphology during early intervals to increasing vacuolation, nuclear alteration, fragmentation, and eventual structural loss at later intervals. The observed association (*p* < 0.001) suggests that Giemsa-stained sternal bone-marrow cytology may serve as a practical and low-cost complementary tool in early-to-intermediate PMI assessment. However, the present findings do not establish predictive accuracy or independent reliability, and marrow morphology should therefore be interpreted alongside other postmortem findings. Further prospective studies incorporating environmental and individual variables, interobserver assessment, and external validation are warranted.

## Data Availability

The raw data supporting the conclusions of this article will be made available by the authors, without undue reservation.

## References

[1] Cockle DL Bell LS. Human decomposition and the reliability of a “universal” model for postmortem interval estimations. Forensic Sci Int. (2015) 253(136):e1–136.e9. 10.1016/j.forsciint.2015.05.018 26092190

[2] Franceschetti L Amadasi A Bugelli V Bolsi G Tsokos M. Estimation of late postmortem interval: where do we stand? A literature review. Biology (Basel. (2023) 12(6):783. 10.3390/biology12060783 37372068 PMC10295266

[3] Biradar G Babu BS Bakkannavar SM Pradeep Kumar G Shaila B. Estimation of time since death from cytoplasm changes of bone marrow cells. J South India Medico-Legal Assoc. (2016) 8(2):85–9.

[4] Sakr MF Abd El-Khalek AM Sameeh N Abouhashem NS Gaballah MH Ragab HM. Estimation of postmortem interval using histological and oxidative biomarkers in human bone marrow. Forensic Sci Med Pathol. (2023) 20(3):910–9. 10.1007/s12024-023-00753-9 37987965

[5] Bonicelli A Giorgetti A Rossi D Bacci M Franchi A Giorgetti R. ForensOMICS approach for postmortem interval estimation: metabolomics, lipidomics and proteomics of human bones. Front Med (Lausanne. (2022) 9:980335. 10.3389/fmed.2022.980335 PMC9803353 36583441

[6] Madea B. Methods for determining time of death. Forensic Sci Med Pathol. (2016) 12(4):451–85. 10.1007/s12024-016-9776-y 27259559

[7] Coe JI. Postmortem chemistry update: emphasis on forensic applications. Forensic Sci Rev. (1993) 5(1):1–25. 10.1097/00000433-199306000-00001 8328447

[8] Henssge C Madea B. Estimation of the time since death. Forensic Sci Int. (2007) 165(2–3):182–4. 10.1016/j.forsciint.2006.05.017 16797901

[9] Salam HA Shaat EA Aziz MH Moneim Sheta AA Hussein HA. Estimation of postmortem interval using thanatochemistry and postmortem changes. Alexandria J Med. (2012) 48(4):335–44. 10.1016/j.ajme.2012.05.004

[10] Tomasian A Jennings JW. Bone marrow aspiration and biopsy: techniques and practice implications. Skeletal Radiol. (2022) 51(1):81–8. 10.1007/s00256-021-03882-w 34398308

[11] Roll P Beham A Beham-Schmid C. Post-mortem histopathological investigations of the bone marrow in forensic medicine: an important issue for both the forensic and clinical pathologist. Forensic Sci Int. (2009) 186(1–3):e17–20. 10.1016/j.forsciint.2008.12.005 19231118

[12] Szűcs D Fejes V Kozma Z Poór VS Sipos K. Sternal aspirate sampling of bacillariophyceae and cyanobacteria in suspected drowning cases. J Forensic Leg Med. (2022) 85:102298. 10.1016/j.jflm.2021.102298 34896890

[13] Amendt J Krettek R Zehner R. Forensic entomology. Naturwissenschaften. (2004) 91(2):51–65. 10.1007/s00114-003-0493-5 14991142

[14] Tattoli L Tsokos M Sautter J Anagnostopoulos J Maselli E Ingravallo G. Postmortem bone marrow analysis in forensic science: study of 73 cases and review of the literature. Forensic Sci Int. (2014) 234:72–8. 10.1016/j.forsciint.2013.10.040 24378305

[15] Babu S Biradar G Bakkannavar S Kumar PG Shaila B. Estimation of time since death from nuclei changes of bone marrow cells. Indian J Forensic Community Med. (2015) 2(4):198–202. 10.5958/2394-6776.2015.00003.X

[16] Madea B Höglund P. Postmortem changes and time of death. Forensic Sci Int. (1989) 42(1–2):213–36.

[17] Wehner F Wehner HD Subke J. Time-dependent expression of heat-shock proteins in human gingival tissue and its forensic application. Int J Legal Med. (1999) 112(6):375–8.

[18] Cattaneo C Di Giancamillo A Gallidabino M Domeneghini C Craig OE. Histological determination of the postmortem interval from human and animal bone. J Forensic Sci. (2009) 54(2):307–15. 10.1111/j.1556-4029.2009.01000.x

[19] Zhang D Li L Chen X Yang L Guo Y. Morphological changes of bone marrow cells after death and its application in estimating postmortem interval. Fa Yi Xue Za Zhi. (2002) 18(4):194–6.

[20] Campobasso CP Di Vella G Introna F. Factors affecting decomposition and postmortem interval estimation: a review of current literature. Am J Forensic Med Pathol. (2001) 22(4):388–92.

[21] Hayman J Oxenham MF. Estimating postmortem interval using bone histology: developing a framework. J Anat. (2016) 229(4):637–46.

[22] Reisinger K Patel J Nguyen J. Postmortem cellular degradation: forensic implications. J Forensic Sci. (2018) 63(6):1780–7.

[23] Prieto-Bonete G Pérez-Cárceles MD Pérez-Martínez C Luna A. Bone diagenesis: useful principles in postmortem interval estimation. Forensic Sci Int. (2015) 247(165):e1–7. 10.1016/j.forsciint.2014.11.003 25549847

[24] Ubelaker DH Shamlou A. Applications of forensic bone histology to postmortem interval estimation. Forensic Sci Int. (2017) 278(417):e1–8. 10.1016/j.forsciint.2017.07.036 28818310

[25] Buchan MJ Anderson GS. Time since death: a review of the current status of methods used in the later postmortem interval. Can Soc Forensic Sci J. (2001) 34(1):1–22. 10.1080/00085030.2001.10757514

[26] Wilson RJ Ubelaker DH. Forensic estimation of postmortem interval using microscopic bone analysis. Microsc Microanal. (2015) 21(6):1314–22. 10.1017/S1431927615015019 26343378

[27] Pounder DJ Stevenson R. Patterned cell autolysis in human tissues: forensic significance. Am J Forensic Med Pathol. (1983) 4(4):298–301.

[28] Zhou C Byard RW. Factors influencing postmortem decomposition of the human body: a review. J Forensic Sci. (2011) 56(1):6–13.

[29] Lee G Go JH. Postmortem changes in soft tissues and implications for postmortem interval estimation. Korean J Pathol. (2012) 46(1):1–9. 10.4132/KoreanJPathol.2012.46.1.1 23109971 PMC3479707

[30] Sato Y Sato K. Histological changes of tissues after death. Nihon Hoigaku Zasshi. (1990) 44(3):238–46.

[31] Tracqui A Borel M Cattaneo C. Biochemical approach to determining postmortem interval: a review. Forensic Sci Int. (1995) 75(3):167–77.

[32] Henssge C Knight B. Postmortem interval estimation in the early phase: review. Forensic Sci Int. (1995) 72(3):173–8.

[33] Zhao J Li L Luo J Wang H. Study of time-dependent autolytic changes in hematopoietic tissues in rats. Chin J Forensic Med. (2008) 23(3):167–70.

[34] Larkin A Evershed RP O'Connell TC. Diagenesis of bone lipids in postmortem interval estimation. J Archaeol Sci. (2014) 52:103–14.

[35] Notter SJ Stuart BH Dent BB. The examination of adipocere formation using differential scanning calorimetry. J Forensic Sci. (2009) 54(3):672–6. 10.1111/j.1556-4029.2009.01023.x 19445027

[36] Vass AA. Odor mortis. Forensic Sci Int. (2012) 222(1–3):234–41. 10.1016/j.forsciint.2012.06.006 22727573

[37] Megyesi MS Nawrocki SP Haskell NH. Using accumulated degree-days to estimate postmortem interval. J Forensic Sci. (2005) 50(3):1–9. 10.1520/JFS2004017 15932096

[38] Pittner S Bugelli V Benbow ME Ehrenfellner B Zissler A Campobasso CP. Forensic thanatobiochemistry: a review. Forensic Sci Int. (2020) 314:110367.

[39] Luna A. Histopathological analysis of postmortem tissues and postmortem interval estimation. Forensic Sci Int. (2016) 258:54–9.

[40] Aly SM Mohamed EA. Postmortem histological changes in rat bone marrow for early postmortem interval estimation. Mansoura J Forensic Med Clin Toxicol. (2012) 20(2):71–82.

[41] Kumar A Verma SK. Time-dependent changes in marrow cytology after death: forensic implications. Egypt J Forensic Sci. (2018) 8:1–8.

[42] Fiedler S Graw M. Decomposition of buried corpses. Forensic Sci Int. (2003) 136(2–3):163–7.

[43] Sorg MH Haglund WD. Forensic Taphonomy in Death investigation. Boca Raton (FL): CRC Press (2002).

[44] Dent BB Forbes SL Stuart BH. Review of burial and decomposition timelines. Forensic Sci Int. (2004) 147(Suppl):S143–7.

[45] Orkin SH Zon LI. Hematopoiesis: an evolving paradigm for stem cell biology. Cell. (2008) 132(4):631–44. 10.1016/j.cell.2008.01.025 18295580 PMC2628169

[46] Morrison SJ Scadden DT. The bone marrow niche for haematopoietic stem cells. Nature. (2014) 505(7483):327–34. 10.1038/nature12984 24429631 PMC4514480

[47] Weissman IL. Stem cells: units of development, units of regeneration, and units in evolution. Cell. (2000) 100(1):157–68. 10.1016/S0092-8674(00)81692-X 10647940

[48] Cline MJ. The hematopoietic microenvironment. N Engl J Med. (1975) 292(17):883–90.

[49] Koury MJ. Erythropoiesis: the cascade of erythroid progenitor differentiation. Semin Hematol. (2016) 53(1):2–6.

[50] Palis J. Primitive and definitive erythropoiesis in mammals. Front Physiol. (2014) 5:3. 10.3389/fphys.2014.00003 24478716 PMC3904103

[51] Manz MG Boettcher S. Emergency granulopoiesis. Nat Rev Immunol. (2014) 14(5):302–14. 10.1038/nri3660 24751955

[52] Italiano JE Jr. Mechanisms and clinical relevance of thrombopoiesis. Hematology Am Soc Hematol Educ Program. (2018) 2018(1):1–8. 10.1182/asheducation-2018.1.1 30504285 PMC6245981

[53] Machlus KR Italiano JE. The incredible journey: from megakaryocyte development to platelet formation. J Cell Biol. (2013) 201(6):785–96. 10.1083/jcb.201304054 23751492 PMC3678154

[54] Lichtman MA. The normal marrow. In: Kenneth K, Marshall AL, Josef TP, Marcel L, Oliver WP, Linda JB, Michael AC, editors. Williams Hematology, 9th Edn. New York (NY): McGraw-Hill (2016).

[55] Zon LI. Intrinsic and extrinsic control of hematopoietic stem-cell self-renewal. Nature. (2008) 453(7193):306–13. 10.1038/nature07038 18480811

[56] Jacobsen SEW Nerlov C. Haematopoiesis in the era of advanced single-cell technologies. Nat Cell Biol. (2019) 21(1):2–8. 10.1038/s41556-018-0227-8 30602765

[57] Bessman JD. Erythroblastic islands and the central macrophage. Blood. (1977) 49(6):855–63.861374

[58] Pinho S Frenette PS. Haematopoietic stem cell activity and interactions with the niche. Nat Rev Mol Cell Biol. (2019) 20(5):303–20. 10.1038/s41580-019-0103-9 30745579 PMC6483843

[59] Scadden DT. The stem-cell niche as an entity of action. Nature. (2006) 441(7097):1075–9. 10.1038/nature04957 16810242

[60] Kondo M Wagers AJ Manz MG Prohaska SS Scherer DC Beilhack GF. Biology of hematopoietic stem cells and progenitors. Nat Immunol. (2003) 4(7):641–8. 10.1038/ni0703-641 12615892

[61] Hartenstein V. Blood cells and their lineages. Dev Biol. (2006) 10(4):195–210.

[62] Bain BJ. Bone marrow biopsy morbidity and mortality. Br J Haematol. (2003) 121(6):949–51. 10.1046/j.1365-2141.2003.04329.x 12786808

[63] Takahashi S Takada A Saito K Hara M Yoneyama K Nakanishi H. Diagnostic significance of histopathology of bone marrow macrophages in forensic autopsies. Leg Med (Tokyo. (2022) 58:102079. 10.1016/j.legalmed.2022.102079 35490477

[64] Castillo P Ussene E Ismail MR Jordao D Lovane L Carrilho C. Pathological methods applied to the investigation of causes of death in developing countries: minimally invasive autopsy approach. PLoS One. (2015) 10(6):e0132057. 10.1371/journal.pone.0132057 26126191 PMC4488344

[65] Neumark E. Sternal marrow biopsy. Postgrad Med J. (1949) 25(288):478–82. 10.1136/pgmj.25.288.478 18148955 PMC2530035

[66] Yadav A Dey A Anuragi RP Kanwar H. Determination of postmortem interval by decomposition changes: an ambiguous phenomenon. Clin Case Rep Rev. (2017) 3:1–3. 10.15761/CCRR.1000327

[67] Galloway A Birkby WH Jones AM Henry TE Parks BO. Decay rates of human remains in an arid environment. J Forensic Sci. (1989) 34(3):607–16. 10.1520/JFS12680J 2738563

[68] Tuljapurkar SR McGuire TR Brusnahan SK Jackson JD Garvin KL Kessinger MA. Changes in human bone marrow fat content associated with changes in hematopoietic stem cell numbers and cytokine levels with ageing. J Anat. (2011) 219(5):574–81. 10.1111/j.1469-7580.2011.01423.x 21923862 PMC3191309

[69] Giles SB Errickson D Márquez-Grant N. Decomposition variability between the scene and autopsy examination and implications for postmortem interval estimations. J Forensic Leg Med. (2022) 85:102292. 10.1016/j.jflm.2021.102292 34839087

[70] Harvey ML Gasz NE Voss SC. Entomology-based methods for estimation of postmortem interval. Res Rep Forensic Med Sci. (2016) 6:1–9. 10.2147/RRFMS.S68867

[71] Findlay AB. Bone marrow changes in the postmortem interval. J Forensic Sci Soc. (1976) 16(3):213–8. 10.1016/S0015-7368(76)71063-6 1022843

[72] Hoffman SB Morrow GW Jr Pease GL Stoebel CF. Rate of cellular autolysis in postmortem bone marrow. Am J Clin Pathol. (1964) 41(3):281–6. 10.1093/ajcp/41.3.281

[73] Sakr MF Abd El-Khalek AM Sameeh N Abouhashem NS Gaballah MH Ragab HM. Postmortem interval estimation using biochemical antioxidant markers and histopathological changes in human bone marrow. Forensic Sci Med Pathol. (2019) 15(4):617–26.

[74] Bansal A Sharma S Verma R Gupta M Singh P. Histopathological evaluation of bone marrow in medicolegal autopsies and its diagnostic relevance. Indian J Forensic Med Pathol. (2022) 3:41–4.

[75] Weustink AC Hunink MGM Van Dijke CF Renken NS Krestin GP Oosterhuis JW. Minimally invasive autopsy: an alternative to conventional autopsy? Radiology. (2009) 250(3):897–904. 10.1148/radiol.2503080421 19244053

